# Improvement After Surgery in a Patient With Prolonged Tetraplegia Due to Cervical Spinal Cord Injury Without Bone Injury

**DOI:** 10.7759/cureus.33420

**Published:** 2023-01-05

**Authors:** Ippei Kitade, Hideaki Nakajima, Hiroki Nakagawa, Ai Takahashi, Akihiko Matsumine

**Affiliations:** 1 Division of Rehabilitation Medicine, University of Fukui Hospital, Fukui, JPN; 2 Department of Orthopaedic Surgery, University of Fukui, Fukui, JPN; 3 Division of Rehabilitation Medicine, Fukui Prefectural Hospital, Fukui, JPN; 4 Department of Rehabilitation Medicine, University of Fukui, Fukui, JPN

**Keywords:** cervical spinal cord injury, neuro-rehabilitation, operative indication, conservative treatment, cervical decompressive surgery, cervical spinal cord injury without radiographic abnormalities

## Abstract

Treatment strategies for patients with cervical spinal cord injury (CSCI) without major bone injury in the acute phase are under debate. For CSCI without major bone injury, conservative treatment is often the first choice owing to the absence of fractures and spinal column instability. However, treatment of CSCI without major bone injury by either surgery or conservative measures remains controversial. We described a case of a 48-year-old man with cervical American Spinal Cord Injury Association Impairment Scale (AIS) grade C tetraplegia as a result of a fall. Computed tomography scan and magnetic resonance imaging revealed no fractures and widespread T2-hyperintense signal changes in the cord centered on C3-4. The paralyzed condition of his lower extremities remained unchanged with conservative treatment for eight months after the injury. Therefore, he underwent decompression surgery eight months after the injury. At two weeks postoperatively, he could transfer and walk using a walker. After discharge, he underwent regular home-visit rehabilitation and gradually improved his physical functions, including gait ability one year postoperatively. We encountered a case in which surgery and intensive rehabilitation eight months after the injury improved motor function. The combination of surgery in the chronic phase and postoperative rehabilitation can therefore improve the outcomes. The message in this paper is by no means a recommendation for "late surgery." However, we suggested that surgical treatment might be an option if the functional improvement is poor, as even quite late surgery can provide functional improvement.

## Introduction

Cervical spinal cord injury (CSCI) without major bone injury (CSCIw) is described as a spinal cord injury without evidence of spinal fracture or dislocation on plain radiography or computed tomography [1-4]. Surgical treatment is required for traumatic CSCI patients, patients with discoligamentous injuries of intervertebral instability, and patients with progression of myelopathy after trauma, but treatment strategies for patients with CSCIw are controversial. Kawano et al. [1] reported that no significant difference was observed in the American Spinal Cord Injury Association (ASIA) motor scores between surgery and conservative treatment groups at two, three, and six months and one year postoperatively in patients with CSCIw. Although conservative treatment is often adopted as the first choice owing to the absence of fractures and spinal column instability, whether some patients deserve to be treated surgically with the aim of significant function recovery needs further investigation.

We describe a patient diagnosed with CSCIw who underwent decompression surgery in the chronic phase. He was previously chosen for conservative treatment; however, this option did not improve his paralysis eight months after the injury. Therefore, although not acute phase, he underwent decompression surgery and postsurgical rehabilitation eight months after the injury and showed drastic improvement in lower limb motor function. We also discussed changes in lower extremity motor function postoperatively that produced the favorable outcomes observed in this patient.

## Case presentation

**Figure 1:**
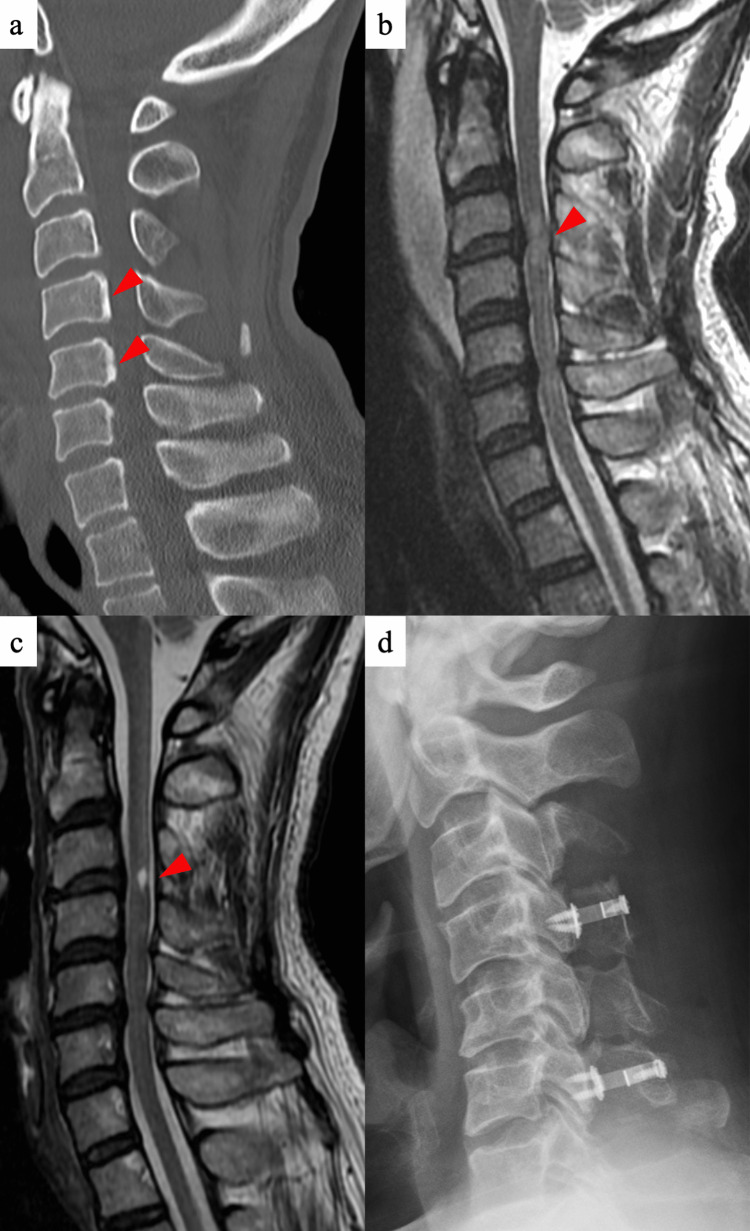
Imaging findings before and after surgery (A) Computed tomography scan after emergency transport; a narrow canal between C4 and C5 (11 mm), ossification of the posterior longitudinal ligament of the segmental type on the posterior walls of C4 and C5, and no fractures. (B) T2-weighted magnetic resonance imaging scan after emergency transport. The spinal cord compression rate was 21%. (C) T2-weighted magnetic resonance imaging scan at eight months after injury. Edema was reduced, and the spinal cord compression rate was 16%. (D) X-ray findings after decompression surgery. The spinal canal enlargement rate was 173%.

A 48-year-old man sustained a fall, resulting in a CSCIw. Computed tomography scan after emergency transport revealed no fractures, but a narrow canal between C4 and C5 (11 mm) and ossification of the posterior longitudinal ligament of the segmental type on the posterior walls of C4 and C5 (Figure 1A). Furthermore, magnetic resonance imaging (MRI) revealed widespread T2-hyperintense signal changes in the cord on C3-4 (Figure 1B). The spinal cord diameter was measured at both the non-compression and injured levels on T1-weighted MRI, and the spinal cord compression rate [1,4] was calculated to be 21%. This patient had no neurological symptoms due to degenerative cervical myelopathy before the injury and had neurological symptoms such as paralysis after the trauma.

Conservative treatment was selected and rehabilitation treatment was started from the day after the injury. The clinical parameters are listed in Table 1. ASIA Impairment Scale (AIS) grade C, modified Ashworth scale (MAS) grade 1 in the bilateral lower limb flexors, and numbness/hypesthesia below T5 were observed 72 hours after the injury. Therefore, he was unable to perform activities of daily living (ADL) normally and spent all day in bed with full assistance (Spinal Cord Independence Measure (SCIM): 8/100 points). He also received general rehabilitation (sitting, standing, and range of motion (ROM) exercises, including muscle strengthening exercises for upper and lower limbs) until seven months after the injury. His motor and sensory functions eight months after the injury did not change substantially compared with those 72 hours after the injury (AIS grade C and numbness/hypesthesia below T5). Additionally, the degree of spasticity changed to MAS grade 3 in the bilateral lower limb flexors and extensors, indicating worsened spasticity, and severe synkinesis of the bilateral lower extremities was observed. He could maintain a sitting posture. His International Stoke Mandeville Wheelchair Sports Federation (ISMWSF) classification was “Fair,” and his SCIM score eight months after the injury was 17/100 points (Table 1). T2-weighted MRI at eight months after the injury revealed myelomalacia on C3/4, and disc protrusion at C3-6 (Figure 1C). Edema was reduced, and the spinal cord compression rate was 16%.

**Table 1:** The clinical parameters from initial onset to 20 months after injury (one-year after surgery) ASIA: American Spinal Cord Injury Association; MAS: modified Ashworth scale; SCIM: Spinal Cord Independence Measure; ISMWSF: International Stoke Mandeville Wheelchair Sports Federation; Bil.: bilateral; L/E: lower extremity.

	Initial onset	Before surgery	After surgery	
		8 months after the injury	17 days after surgery	1 year after surgery
Numbness/hypesthesia	++	++	+	+
	Below T5	Below T5	Below T5	Below T5
ASIA Impairment Scale	C	C	D	D
Gait ability	Impossible	Impossible	With a walker (10 m)	With a walker (100 m)
MAS	1	3	1	1
	Bil. L/E flexors	Bil. L/E flexors	Bil. L/E flexors	Bil. L/E flexors
		Bil. L/E extensors	Bil. L/E extensors	Bil. L/E extensors
SCIM (points)	8	17	20	27
ISMWSF classification	Fair	Fair	Good	Good

He underwent C4-6 open-door laminoplasty eight months after the injury (Figure 1D). The spinal canal enlargement rate was 173%.

**Figure 2:**
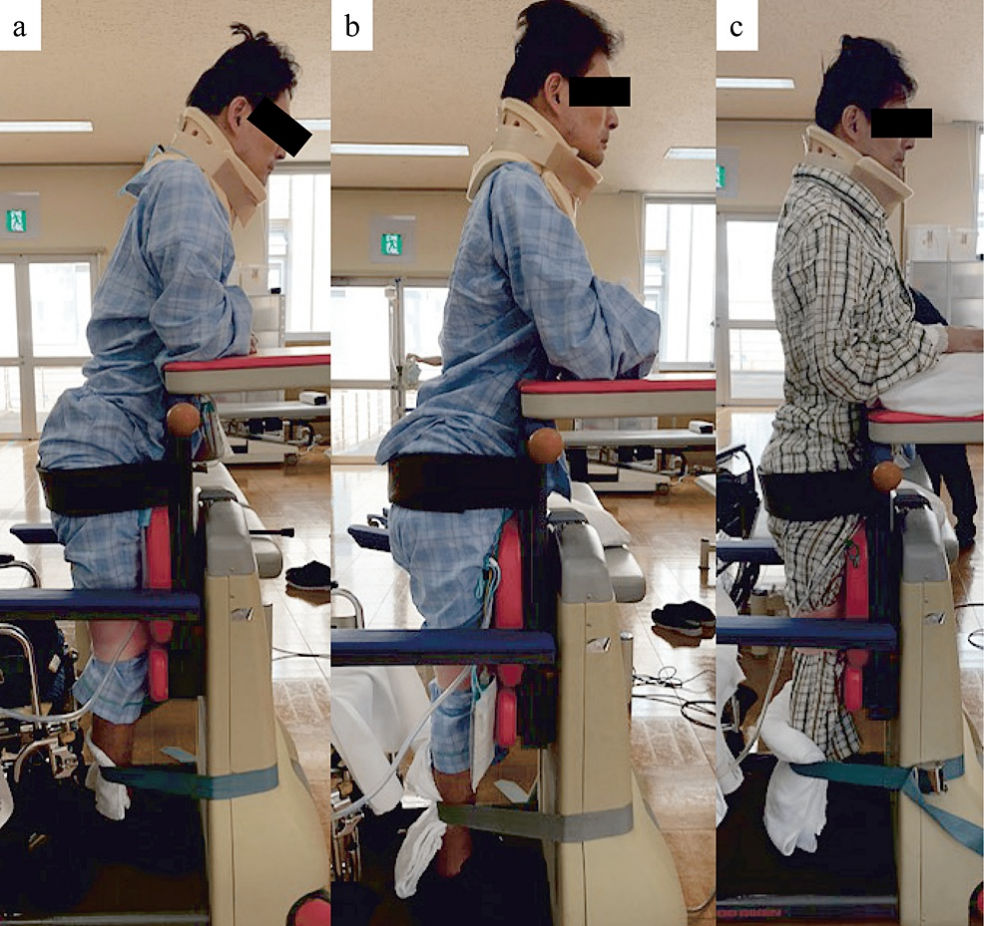
Short-term progress of the standing posture (A) Postoperative day two: Standing posture indicated hip and knee joint flexion; however, foot-flat was not observed (heel-off; plantar-flexed position of the ankle joint). (B) Postoperative day six. (c) Postoperative day 10: The patient was able to attain foot-flat and stand positions in the midline position.

On postoperative day one, he started undergoing a rehabilitation program comprising sitting and standing exercises, including ROM and muscle strengthening exercises for both legs. However, he was unable to attain a standing position owing to the high spasticity of his lower limbs (particularly hip and knee flexors, and ankle plantar flexors). Therefore, he underwent the standing exercises for continuous stretching using three-point support (hip, knee, and ankle) and body weight. His standing posture at two days postoperatively indicated hip and knee joint flexion; however, foot-flat was not observed (heel-off; plantar-flexed position of the ankle joint). On postoperative day 10, the muscle tone of the hip and knee flexors, including ankle plantar flexors, decreased (each muscle: MAS-1), and he was able to attain foot-flat and stand positions in the midline position (Figure 2). Furthermore, he could stand using a walker without three-point support and had no buckling. Gait exercises using a walker were started 11 days postoperatively. At 14 days postoperatively, he could walk 10 m under moderate assistance with a walker (AIS-D), and transfer with mild assistance, for example, between the bed and the wheelchair. However, no improvement was observed in his upper extremity function compared with his lower extremity function. His SCIM score had improved to 20/100 points at 14 days postoperatively. Therefore, he was discharged at 17 days postoperatively and underwent regular home-visit rehabilitation. Other clinical parameters at 17 days postoperatively are listed in Table 1.

At the final, one-year follow-up evaluation (Table 1), he had numbness in both legs, but he could transfer under mild assistance. Although he could walk 100 m using a walker (AIS-D), he did not usually walk at home. MAS of the hip and knee flexors, and ankle plantar flexors were maintained (MAS-1). Subsequently, he was taking a positive slant on intrathecal baclofen or Botox therapy for spastic muscles, and no reduction in ADL was observed at one year postoperatively (SCIM: 27/100 points). Although no major change was observed in the SCIM score, reduced spasticity resulted in reduced assistance requirement during transfer movement, which extended the time spent in the wheelchair-sitting position.

Informed consent to publish this case report was obtained in writing from the patient. Our institution does not require ethical approval for case reports.

## Discussion

Acute treatment of CSCIw remains controversial. Song et al. [5] concluded that surgical management of subaxial acute traumatic CSCI without fracture or dislocation improved neurological status and prevented delayed neurological deterioration in patients. Previous studies have also reported that conservative treatment should be selected for patients with AIS grade B/C and a spinal cord compression rate of <20% [1]. Additionally, no significant difference has been observed in functional recovery between surgical and conservative treatments in cases with a spinal cord compression rate of ≥20%. Nakajima et al. [4] recommended surgical treatment for acquiring walking ability in patients with a spinal cord compression rate of ≥ 33.2% and low ISMWSF grade at the subacute phase. Regarding the timing of surgery, some reports on CSCIw have recommended surgery within 24 hours of injury [6,7]. Surgery within 24 hours is recommended for acute spinal cord injury and central cord syndrome, but the neurological prognosis of CSCIw patients can be difficult to determine in the acute phase. The message in this paper is by no means a recommendation for "late surgery." However, we suggested that surgical treatment might be an option if the functional improvement is poor, as even quite late surgery can provide functional improvement [8,9].

This patient had no neurological symptoms due to degenerative cervical myelopathy before the injury and had neurological symptoms such as paralysis after the trauma. The spinal cord compression rate after emergency transport was approximately 20%, and the ISMWSF classification at the time of injury and eight months after the injury was “Fair.” However, the patient’s paralyzed condition of the lower extremities remained unchanged with conservative treatment for eight months after the injury. Our case report appears to be the first to show improvement in paralysis by surgery and postoperative rehabilitation treatment for CSCIw despite the chronic phase. At two weeks postoperatively, the patient could transfer and walk using a walker. After discharge, he underwent regular home-visit rehabilitation and gradually improved his physical functions, including gait ability one year postoperatively. Surgical treatment may be aggressively performed on a patient who had prolonged tetraplegia due to CSCIw. Thus, it is important to predict the prognosis from clinical imaging and physical motion findings in the acute or subacute phases to determine the ideal treatment method [1,3,4]. However, there is still insufficient evidence on prognosis prediction using findings in the early phase of the injury. It will also be necessary to clarify the mechanism by which surgery in the chronic phase improved paralysis.

## Conclusions

We suggested that there are cases of chronic CSCIw in which surgical treatment should be performed. In this case, the combination of surgery in the chronic phase and postoperative rehabilitation can therefore improve the outcomes. As a limitation, our presentation is only a case report. Hence, future studies with larger patient populations and further quantitative parameters, such as imaging and physical findings, are warranted to determine the ideal treatment method.
